# Revisiting the Wood Criteria for Frontotemporal Degeneration Pedigree Classification: 10 Years Later

**DOI:** 10.1002/alz.087652

**Published:** 2025-01-03

**Authors:** Laynie Dratch, Julia Kwiecinski, Vivianna M Van Deerlin, EunRan R Suh, Allyson Hinks, David J Irwin, Lauren Massimo, Sara Manning Peskin, Corey T McMillan

**Affiliations:** ^1^ Frontotemporal Degeneration Center, University of Pennsylvania, Philadelphia, PA USA; ^2^ Penn Frontotemporal Degeneration Center, University of Pennsylvania, Philadelphia, PA USA; ^3^ University of Pennsylvania, Philadelphia, PA USA; ^4^ Center for Neurodegenerative Disease Research, Perelman School of Medicine, University of Pennsylvania, Philadelphia, PA USA; ^5^ University of Pennsylvania, School of Nursing, Philadelphia, PA USA; ^6^ Penn FTD Center, University of Pennsylvania, Philadelphia, PA USA

## Abstract

**Background:**

Previously, the Penn Frontotemporal Degeneration (FTD) Center developed and validated criteria to stratify pedigrees of patients with FTD by likelihood of identifying a genetic etiology (Wood, JAMA Neurol., 2013). Pedigrees were classified as high‐risk, medium‐risk, low‐risk, apparent sporadic, or unknown significance. Genetic testing for the three most common genes that cause FTD (C9orf72, MAPT, GRN) was completed to assess the detection rate of genetic cases per category. Given advances in FTD genetics, the current study aims to cross‐validate this pedigree classification criteria in an independent cohort and determine the extent to which consideration of newly identified disease‐associated genes increases the detection rate.

**Method:**

First, pedigrees in the initial cohort (n = 306) were reclassified when appropriate based on updated family history information. Next, we evaluated whether more comprehensive genetic analysis altered detection rates in the initial cohort. Then, we applied the Wood criteria to an independent cohort of pedigrees of probands with FTD‐spectrum disorders (n = 400) and assessed detection rate with testing for exclusively C9orf72, MAPT, and GRN, and then with broader sequencing.

**Result:**

In the initial cohort, genetic analysis beyond C9orf72, MAPT, and GRN identified genetic etiologies in six additional pedigrees, five of which were in the high‐risk group, raising the overall cohort detection rate from 15% to 17%. In the independent cohort, when only C9orf72, MAPT, and GRN were analyzed detection rates were approximately 18% (72/400) overall, 67% (38/57) in the high‐risk group, 29% (15/51) in medium‐risk, 9% (6/70) in low‐risk, 3% (4/135) in apparent sporadic, and 10% (9/87) in unknown significance. Broader sequencing identified two additional genetic cases in the high‐risk group and one in the apparent sporadic group, increasing the overall cohort detection rate from 18% to 19%.

**Conclusion:**

These results reiterate the importance of gathering a detailed neurologic family history in genetic risk assessment for FTD. Given the emergence of gene‐specific clinical trials, we suggest that using this cross‐validated pedigree classification criteria provides an accurate first step in determining the likelihood of a genetic diagnosis. Genetic counseling and comprehensive testing should be routinely offered in clinical care for all patients with FTD, regardless of risk stratification.

